# What contemporary viruses tell us about evolution: a personal view

**DOI:** 10.1007/s00705-013-1679-6

**Published:** 2013-04-09

**Authors:** Karin Moelling

**Affiliations:** 1 Max Planck Institute for Molecular Genetics, Ihnestr 63-73, 14195 Berlin, Germany; 2 Institute for Medical Microbiology, Gloriastr 32A, 8006 Zürich, Switzerland

## Abstract

Recent advances in information about viruses have revealed novel and surprising properties such as viral sequences in the genomes of various organisms, unexpected amounts of viruses and phages in the biosphere, and the existence of giant viruses mimicking bacteria. Viruses helped in building genomes and are driving evolution. Viruses and bacteria belong to the human body and our environment as a well-balanced ecosystem. Only in unbalanced situations do viruses cause infectious diseases or cancer. In this article, I speculate about the role of viruses during evolution based on knowledge of contemporary viruses. Are viruses our oldest ancestors?

## Introduction

New technologies have changed our understanding of viruses throughout the last ten years. Viruses are not primarily pathogens, which is a biased view based on the history of medicine. Most viruses do not cause diseases. Viruses cause diseases if a well-established equilibrium, which evolved over billions of years, gets out of balance.

A glance at some numbers may support the notion that viruses are much more than just pathogens. There are 10^33^ viruses on our planet, about 10 times more than bacteria. There are only about 10^9^ human beings – a small minority, which makes us the invaders in the viral world, not vice versa. They are present in the oceans, 10^12^ per ml [96, 97], in the soil, abundant in plants, and inside the human body. Healthy humans consist of about 10^13^ cells and harbor 10^14^ to 10^18^ bacteria [101] and an unknown number of viruses. Bacterial information is our second genome, with a total genetic complexity about 100 times greater than that of our own genome. We are 99 % bacteria – with respect to the total genetic information of our body. Viruses may be our third genome [40, 112]. We harbor about 1.5 kg of bacteria in our guts – 1,500 different types. Viruses are also a major component in our guts. Two hundred types have been detected in human gut samples based on similarities to known viruses [80]. Archaea and fungi are also present in our guts [28, 80, 84, 85]. Thus, we are a superorganism as well as a complicated ecosystem [41]. Phages or bacteriophages are viruses of bacteria. They can lyse bacteria, which gave them their name. A gut microbial gene catalogue is being established by ongoing metagenomic sequencing [85, 112]. It was a surprise to learn that, instead of a constant battle going on between viruses and cells fighting for dominance in our guts, the two are actually in a well-balanced equilibrium [40].

It is the purpose of this article to discuss what we can learn from contemporary viruses about their potential role during the history of life and evolution – apart from causing diseases. Their contribution to the development of life, genome composition, genetic diversity, our environment, and our body will be evaluated here.

## RNA and viroids

The beginning, when life started, was an RNA world, as this is widely accepted today [37, 41, 42, 44]. We do not really know how the first nucleotides, the building blocks of RNA, arose. They are difficult to synthesize. Some black smokers – hydrothermic vents with extreme temperatures and temperature gradients from 400 degrees to cold temperatures – at the bottom of the oceans may have allowed the synthesis of RNA. Clay could have supplied catalytic help. Rocks composed of metal-rich granite helped life to evolve. Energy was supplied from chemical reactions, not directly from sunlight, because 200 m below sea level the world is dark.

RNA evolved to catalytic oligonucleotides, known today as ribozymes. Catalytic RNA can cleave and join RNA molecules *in vitro* in laboratory experiments. It can replicate, mutate and evolve [60]. Plant pathogens known as viroids reflect properties of the early RNA world. Viroids are ribozymes. They are widespread in plants and can be a threat to many crops [35]. The route of transmission has been attributed to knives used for harvesting – reminiscent of contaminated needles in human viral diseases. They look like remnants of a pre-protein world, since instead of coding for proteins, they consist of non-protein-coding naked RNA without protein coats. They are small – only a few hundred nucleotides in length – and their single-stranded RNA is often folded in a hairpin-loop structure, which protects against environmental threats [22–24]. The absence of coding information suggests that the viroids have structural information. It has been suggested that a viroid may have entered the human body and developed into a human virus, hepatitis delta virus HDV [99], which may have acquired genetic information for a protein from the host, because HDV antigens are related to a human protein [12, 17, 18, 36]. HDV is the only virus known to be a catalytic ribozyme and pathogen in humans. Why is there only one? Recently, catalytic ribozymes have been identified in many organisms: bacteria, archaea, carnation flowers, fungi, amoebae. They are apparently ubiquitous and may play a role in splicing [44].

The next progress in evolution may have been the plant viruses, such as tobacco mosaic virus or related viruses coding for few proteins [31]. They are extremely stable with rod-like structures and can pass through our gastrointestinal tract without degradation [112]. They are even secreted in an infectious form and may infect plants; 10^9^ viruses per gram of plant material can be ingested by humans, and similar amounts are excreted. Infectious pepper mild mottle virus (PMMV) belongs to this widespread group of plant viruses and is even found in chili sauce without being pathogenic for people [112]. Many plant viruses do not code for their own replicase, using instead the cellular RNA replicases, which were probably the first and oldest polymerases [55].

Almost all plant viruses are small. Yet the total sequence information contained even in small RNA viruses is immense. There is more genetic information than is being exploited in all biological systems on our planet [4, 5]. A mixed cloud of genomes, a quasispecies, must have been important initially [4, 5]. For the initial genetic information or variability of a simple small RNA molecule to further increase, the molecule could not just increase its length, as it would become more unstable. Instead, several RNA molecules may have accumulated in some kind of protective compartment. This is reflected in viruses with segmented genomes today. Segmentation of RNA is detected today in RNA viruses such as influenza viruses [31].

## From RNA to proteins

**Fig. 1:**
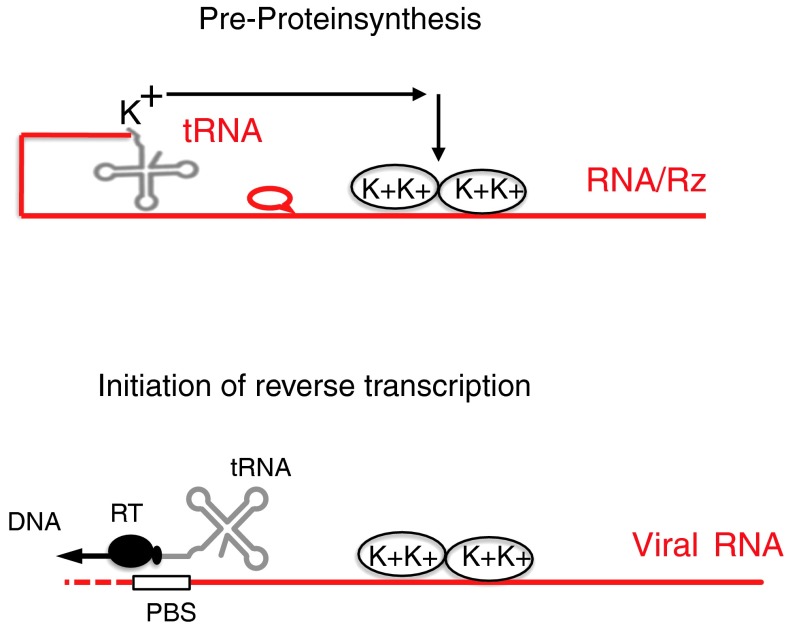
**Pre-protein synthesis. (top)** Early protein synthesis may have started with a short target RNA or ribozyme (Rz), binding to basic amino acids (K + lysine). Some plant viral genomes end with a tRNA, shown as fold-back – perhaps the future tRNA. The basic amino acids protected and enhanced the catalytic activity of the ribozyme. (**bottom**) This is reminiscent of today’s initiation of replication of retroviruses, which have an initiation complex similar to the early protein synthesis complex. The tRNA binds a protein, reverse transcriptase (RT) and basic proteins, the nucleocapsid proteins (NC), which melt or match the RNAs. Initiation of replication is reminiscent of primitive protein synthesis. RNA is shown in red and DNA in black. PBS, primer-binding site

**Fig. 2:**
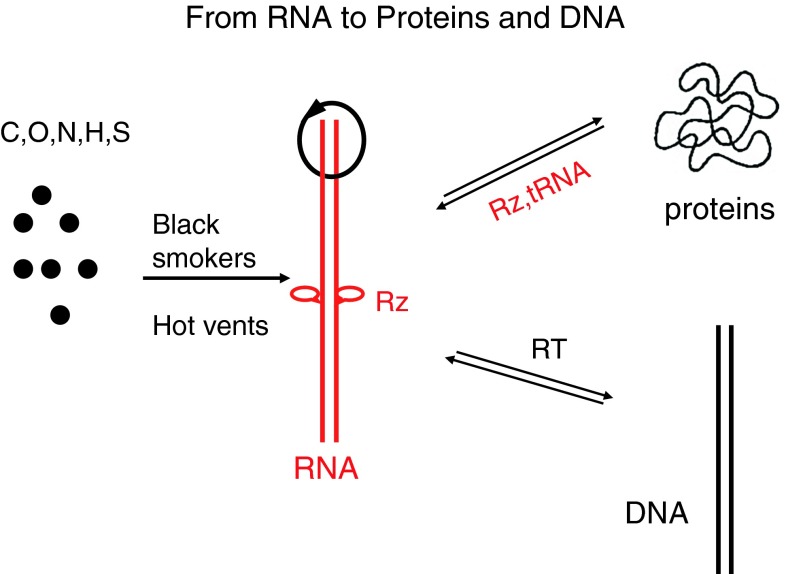
**From RNA to proteins and DNA**. Molecules may have surrounded black smokers or hot vents in the oceans and led to the formation of ribozymes or viroids, which can replicate, cleave and fuse, and evolve, which are hallmarks of life. The protein synthesis machinery consists of ribozymes and basic proteins, ribonucleoproteins (RNPs). Reverse transcriptase (RT) achieved the transition from RNA to DNA

The catalytic activity of ribozymes or deoxyribozymes is limited compared to protein enzymes. They also replicate poorly [60]. Some RNA synthesis can occur by non-enzymatic mechanisms [50]. However, proteins accelerated the reactions. Proteins may have developed next. How did they arise? Viruses may tell us. RNA viruses developed strategies to protect the ends or their RNA; some of them have structures like tRNAs. tRNAs could fold back and bind to a ribozyme or target RNA and transfer individual amino acids. This could have been the beginning of peptide synthesis (Fig. 1). DNA was not yet needed. A complex of ribonucleoproteins (RNPs) and ribozyme RNA then gave rise to ribosomes. “Ribosomes are ribozymes” [74, 93]. Even today, the catalytic activity of ribosomes is provided by a ribozyme, and about half of the 100 ribosomal proteins today are RNPs with scaffold function [93]. RNPs accelerate the catalytic activity of ribozymes [77] (Fig. 2). RNPs are present in many RNA viruses, such as the nucleocapsid protein NCp7 in HIV and the nucleoprotein NP in influenza virus [31]. Often they are flexible, with two clusters of the basic amino acids arginine and lysine, as is found in NCp7. These basic proteins combine many functions. They are RNA binders and neutralize its charge, protect against nucleases, bind cooperatively as chaperones or matchmakers, melt and unwind template RNAs for efficient transcription and unfold rigid tRNAs to serve as primers for initiation of replication of retroviruses. Most importantly, nucleocapsids have been shown to strongly activate ribozymes. They can stimulate their catalytic activity up to 1000-fold [21, 77]. Thus, basic amino acids or peptides may have improved ribozymes and speeded up evolution (Fig. 1). Today 80 % of the ribosomal proteins have a positive charge and bind to the RNA. Yet the basic amino acids are not the simplest ones, which would be alanine and glycine. RNA enzymes may have evolved to protein enzymes later. There are examples suggesting that RNA developed to proteins, e.g., the antiviral defense mechanism from RNA-based siRNA to protein-based interferon [63], but this is speculative.

## From RNA to DNA

**Fig. 3:**
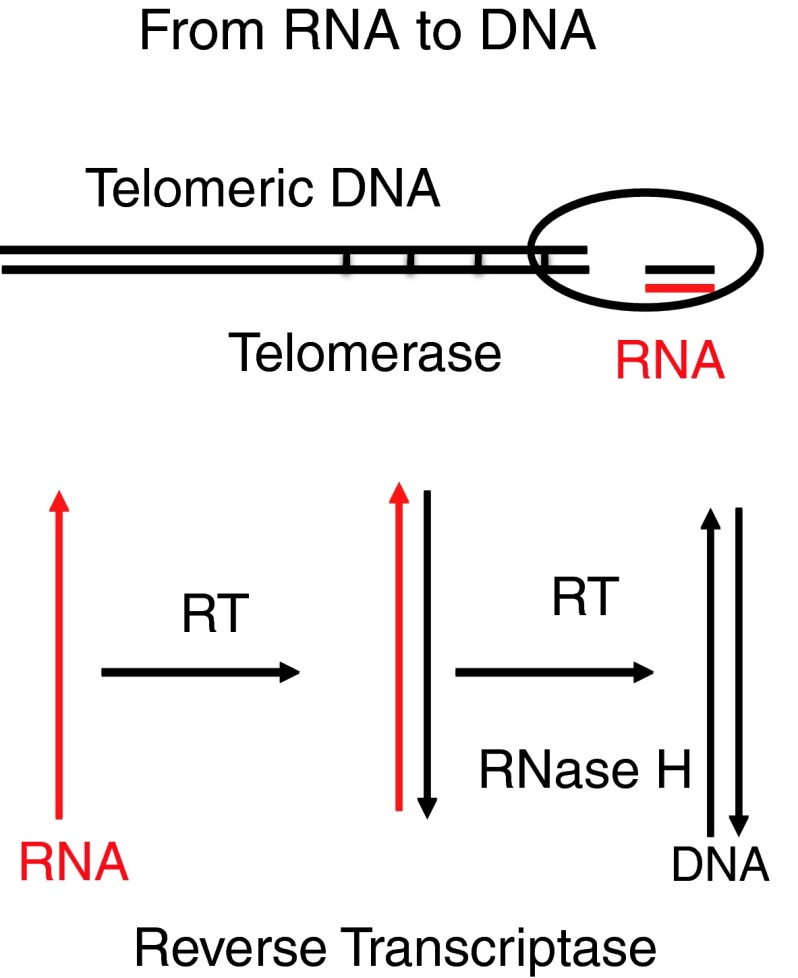
**From RNA to DNA. (Top)** This reaction is performed by the telomerase in every embryonic eukaryotic cell and in tumor cells at chromosomal ends. The telomerase is an RNP and copies a simple stretch of RNA into DNA up to 1000-fold. (**Bottom**) Reverse transcriptase (RT) copies RNA into an RNA-DNA hybrid and into a double-stranded DNA, supported by ribonuclease H (RNase H), which removes the RNA in RNA-DNA hybrids and RNA primers

How and when the transition from RNA to DNA occurred is a matter of speculation. DNA is much less multifunctional than RNA. DNA has a long-term memory and stabilizes genetic information, in contrast to the variable RNA. Deoxyribonucleotides may have formed without or with enzymes, such as a ribonucleotide reductase. In a protein world, the transition from RNA to DNA can possibly be witnessed today in embryonic and cancer cells, as well as in the replication of retro- and pararetroviruses. This transition occurs at the ends of chromosomes by telomerases in embryonic mammalian cells [8, 9] and in tumor cells [45]. The telomerases copy a short RNA sequence into DNA over and over again (Fig. 3). Telomerase is a specialized reverse transcriptase, a complex of an RNA-dependent DNA polymerase and an endogenous RNA. The RNA contains limited information, consisting solely of repeats of a few ribonucleotides, leading to the repeats at the telomeres of TTAAGGG or similar sequences in other species, which are repeated up to 1000 times, depending on the organism. Telomeric sequences protect genomes from losing information by shortening during replication. Watson and Crick already foresaw this problem when they first described the DNA double helix [111]. In adult cells, where the telomerases are inactive, insertion of telomerases can reverse aging and prevent death, as biotech companies are actively investigating. Telomerases can be detected in almost all forms of life. The telomeric structure of RNA/DNA in pseudoknots may be reminiscent of the pseudoknot structure of the RNA of some ribozymes. Whether there is an evolutionary relationship is not clear but may be worth analyzing [25, 29, 43, 44, 79].

A relative of telomerase is reverse transcriptase (RT), which copies complex RNA into DNA. It is a hallmark of retroviruses but present in many organisms independent of retroviruses [57, 58, 90] (Fig. 3). The name is historical and based on the discovery of retroviral replication. The name “reverse” refers to the Central Dogma, the way from DNA to RNA to proteins, coined by Sir Francis Crick, which may not have been meant as dogmatically as it sounds [20]. Reverse transcription from RNA to DNA was unexpected, even though it was probably the oldest direction of flow of genetic information during evolution. Howard Temin, who was trying to demonstrate its existence, was surrounded by skepticism even among his own coworkers. Finally he and David Baltimore discovered the RT and received the Nobel Prize in 1975. In the pre-protein world there were precursors of RTs, the group II introns, consisting of self-splicing RNA, which are mobile ribozymes that can invade DNA. They occur in bacteria, archaea and phages [57]. They are relatives of retrotransposons and may represent an evolutionary link between the RNA ribozymes and the transition to DNA before a protein RT evolved. The “retroelement hypothesis” suggests that group II ribozymes differentiated into retroelements [57, 64]. One can still find this link between ribozymes and introns today [57]. The presence of two RNA genomes per retrovirus particle may be reminiscent of the two ribozymes required for ribozyme replication [60]. There are RTs in many organisms: in bacteria 600 Mio years ago, in archaea [84], bacteriophages, plants, insects and in retroelements (REs) of eukaryotes [31, 42, 61, 90, 110]. Rudimentary retroviroids exist in plants [100, 104].

REs express RNA, which is reverse transcribed and reintegrated. The simplest effect would be gene duplication. Thus, RT helped the genomes to grow. Mutations may have created new information instead of duplicating genes. Retrotransposons code for their own RTs; other transposable elements lacking an RT can use one that is provided *in trans* from a retrotransposon [59]. There is an RNA-DNA transition described for a plant viroid. A viroid in carnations ended up as DNA, perhaps by a reverse transcription mechanism [22, 104].

RTs are found in bacteria, where no retroviruses or retrophages are known. Perhaps the enzyme was left over from ancient retrovirus infections. Alternatively, RT could be a precursor of retroviruses, since it is not always linked to retroviruses. There are even RT-related sequences in phage genomes, yet no retrophages are known. However, RT has an important function there: it can increase the diversity of the phage tails, allowing phage transmission to new hosts, which is an unexpected function [90]. RTs in bacteria can act against phage infection or promote it. Dozens of novel kinds of RTs have been discovered in bacteria, most of them with unknown consequences [90]. RTs are even part of the archaeal, bacterial and mammalian defense systems CRISPR against invaders such as viruses and phages [42, 49, 92]. Reverse transcription affected the formation, content and structure of most eukaryotic and also some prokaryotic genomes, even without viruses [58]. It must have played a major role in evolution [56]. The transition from RNA to DNA has been discussed previously, where it was attributed to viruses. According to that hypothesis, viruses invented DNA, which preceded the formation of the three domains of life [37]. We speculate, viruses invented cells.

In the case of retroviruses, the RT is fused to a ribonuclease H, RNase H, a hybrid-specific nuclease, which cleaves the RNA moiety in RNA-DNA hybrids. It plays an important role in retrovirus replication by generating and removing RNA primers and the RNA template after it has been copied into a DNA strand. Removal of the RNA then allows synthesis of the second DNA strand [47, 70, 73]. Indeed, the RT cannot only copy RNA into DNA but also DNA into double-stranded DNA, which can integrate into the host genome. Thus, the RT appears to be an important evolutionary link between RNA and DNA. Instead of being retrovirus-specific, RNases H specialized in removing RNA primers, upon which even cellular DNA synthesis almost always depends. This may be left over from the RNA world. RNases H may contribute to genome stability by removing misincorporated single ribonucleotides from the human genome, which can cause diseases [70]. RNase H structures are among the five oldest protein structures conserved during evolution, even though they differ in primary sequences. Integrases and the antiviral RNA silencing enzyme Argonaute 2 also belong to this family [70].

The RNase H is a good example of a gene acquired by ancestors of today’s retroviruses. Gene sequence analysis suggests that, after a possible duplication of an RNase H domain, one copy degenerated and lost its enzymatic activity and became a linker. The second copy is the functional enzyme. Thus the RT is linked to an enzymatically active RNase H domain via a degenerated RNase H as linker [70]. The RNase H is a good example for increase of genetic diversity by gene duplication.

Also retrotransposons code for an endonuclease besides the RT and integrase [59].

The specificity of an enzyme for RNA or DNA is not absolute. In the laboratory, the choice of divalent cations needed for enzyme activity allows changing its specificity for RNA or DNA [70].

In some respects, retroviruses resemble DNA phages, since both can integrate their DNA as DNA proviruses or prophages into the host genome. Phages more often persist as episomal plasmid DNA in the bacteria. The prophages can be activated to the “lytic” cycle, leading to lysis of the bacteria as a stress response to extreme conditions such as shortage of nutrients as a result of overgrowth. This makes food available for other organisms as sediments in the sea. Phages regulate population densities of bacteria by cycling, killing and regrowth of bacteria. DNA phages are very diverse and abundant. Where did they come from? They must have come once upon a time from an RNA world. In bacteria, there are a few footprints left, such as significant numbers of RTs and sequences resembling those of telomerases [90, 109, 110]. Have retrophages never existed or did they disappear?

The viruses of archaea pose even more questions about their origin. They are almost all double-stranded DNA viruses. This may not be so surprising because of the stability of DNA under the extreme environmental conditions of the host [84]. Archaea share properties with bacteria and eukaryotes [42]. RT, retroelements, and the defense system CRISPR against DNA viruses have all been described in archaea and their viruses, suggesting a role in the transition from RNA to DNA in the tree of life [42, 49, 84, 92].

## From Viruses to cells

Retroviruses integrate into a preexisting cellular DNA. They need a target DNA. If the RT was a major factor in evolution, then one might speculate that retroviral DNA proviruses may have used the DNA of non-integrating pararetroviruses as a target. Pararetroviruses have an incomplete DNA genome but also replicate via an RT, which explains their name. Their DNA does not normally integrate into a preexisting DNA; it may perhaps have been a target for integration of retroviral DNA. The pararetroviruses include hepatitis B virus (HBV), the foamy viruses, and cauliflower mosaic virus in plants [31]. A link between RNA and pararetroviruses has been described as retroviroids, suggesting a developmental relationship [104]. HBV replicates to a double-stranded DNA in a core. Then, a viral RNA copy leaves the core as pregenomic viral RNA, which is reminiscent of mRNA being released from the nucleus into the cytoplasm for protein synthesis. Could the core of HBV be a precursor of the cellular nucleus? Among other possibilities, poxviruses have been described as precursors of cellular nuclei because they replicate in the cytoplasm [105]. The genetic flow of HBV resembles the one that occurs inside the cell today, from DNA to RNA to proteins, the Central Dogma [20]. HBV DNA does not normally integrate during replication. Doing so could lead to hepatocellular carcinoma (HCC) [31].

Where did the cell come from? Could viruses have developed into cells? Could simple lipid bags have been precursors of cells? There are plant viruses with simple lipid coats which may be ancient [35]. Small molecules could move in and out; bigger ones could accumulate inside. They can split into two as required for division [14].

Thinking of a cell as a big virus is very speculative. Yet this idea may be supported by the recent discovery of giant viruses [11, 75, 86, 87], which appear to be a missing link between viruses and bacterial cells. Giant viruses were initially misinterpreted as bacteria and overlooked. They indeed mimic bacteria and are therefore also called mimiviruses. Giant viruses contain some ribosomes and tRNAs but do not synthesize proteins. Do they reflect the evolutionary beginning of a cell with precursors of a protein synthesis apparatus or degeneration of a more complex cell?

**Fig. 4:**
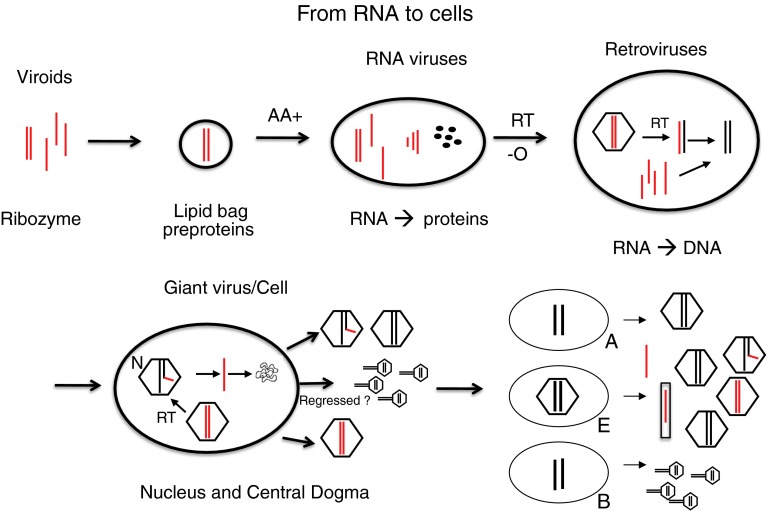
**From RNA to cells: the putative role of viruses during evolution, viruses first.** Ribozymes or viroids, perhaps in lipid bags, may have bound basic amino acids (AA+) and formed peptides (black dots), which stimulated ribozyme activities and became important multifunctional components in all RNA viruses. Self-assembling viral core structures, RNA polymerases and the RT leading to DNA, may have formed. Pararetroviruses and retroviruses used the RT to make DNA, which integrated into other DNA and helped to build up genomes. Up to 50 % retroelements are detectable today in humans. Perhaps something like giant viruses evolved into cells, with bacterial (B), archaeal (A) or eukaryotic cells (E) shedding DNA or RNA viruses. Small tailed structures symbolize phages, which are the fastest replicating and most diverse species, perhaps before separation of A,E and B. Paretroviruses are indicated by incomplete DNA genomes with RNA primers

Mimiviruses were isolated from sewage or water from cooling towers or seawater, where amoebae exist as their host. Phagocytic cells and amoebae separated about 800 Mio years ago. They both contain giant viruses, suggesting that they were both previously infected, although this is a matter of discussion, because they could have been infected independently [102, 103]. They are covered with collagen fibers, which may be a primitive mechanism to trigger their uptake by amoebae. Perhaps these fibers are related to those of bacteria or even hair. There is another aspect in which giant viruses resemble bacteria: They harbor virophages. One of them is called Sputnik [11]. Cafeteria roenbergensis virus (CroV) also harbors a virophage, Ma virus, which has integrated its DNA into the host genome just like a transposable element [33]. Giant viruses are so unique that they have even been discussed as a fourth kingdom in addition to bacteria, archaea and eukaryotes [75]. Giant viruses may have developed into bacteria – or bacteria regressed to viruses. Perhaps giant viruses are dead-end branches in the tree of life? Additional giant viruses will certainly be discovered, since they are abundant, with about 10^8^ viruses per ml in the ocean [102, 103]. They are also important for our environment. Algae blooms, called red tides, that build up in oceans during hot summers are terminated by giant viruses, leading to recycling of the nutrients [102, 103]. The development from viruses to cells as hypothesized here, with a prominent role of RT in the transition from RNA to DNA, is summarized schematically in Fig. 4.

## Horizontal gene transfer

Virus infection of a cell is a very efficient way to supply novel genetic information. A viral infection is more efficient in generating genetic diversity than mutations of cellular genes. The process of a virus acquiring novel genes and introducing them into a cell is described as horizontal gene transfer (HGT). Examples of well-studied viruses that supply novel genes to the host are the oncogenic retroviruses. They can pick up cellular genes, which are modified by high viral mutation rates, before being supplied as oncogenes to a new host cell. Many of them lead to a growth advantage for the recipient cell, which is a hallmark of cancer [45]. Other genes may not be as easily detectable. There are natural oncogenic retroviruses such as bovine and cat leukemia viruses, while others have been selected for and isolated only under laboratory conditions. Well-studied oncoproteins include the kinases Src and Raf, which are involved in many signal transduction cascades and the transcription factors Myc and Myb, which regulate many other genes [26, 71]. About 100 oncogenes are known [31, 45]. This is a surprisingly small number. Overexpression of these genes by strong viral promoters can contribute to their oncogenic potential.

One might expect that HGT by retroviruses could be observed today if we looked at the ongoing retroviral pandemic, the spread of HIV. So far, oncogenes transmitted by HIV have not been described in people. This would have been an interesting large-scale natural experiment for finding new oncogenes or for determining the frequency of cellular gene transfer. About 10^15^ HIV particles are produced daily in the whole HIV-infected world population (30 Mio people with an average of 10^5^ virions/ ml blood and 5 liters of blood). Why does HIV not catch and transmit oncogenes? It must be of a disadvantage. Furthermore, HIV is sometimes a lytic virus, in which case there would be no time for an oncogene to manifest itself inside the cell. HIV can also chronically infect host cells, yet no known oncogenes have been detected. Perhaps HIV is so complex, using the three reading frames and splicing, that acquisition of an oncogene would lead to disruption of essential viral genes. Yet simpler animal leukemia viruses pick up oncogenes, eliminating indispensible genes such as the RT, which is then supplied *in trans* by complementation from a helper virus. A relative of HIV did indeed succeed in expressing an oncogene, the human T-cell leukemia virus HTLV-I, which codes for a transactivator, Tax. Tax can turn on an autocrine loop for growth factor signaling – one step towards cancer. This leads to adult T-cell leukemia, which is endemic in Japan. However, Tax is not a typical oncogene. It has no known proto-oncogene as a cellular homologue, and the mechanism of uptake is not typical for an oncogenic retrovirus because the virus remains fully replication-competent and is independent of a helper virus for its replication [31]. HGT by retroviruses under today’s conditions does not appear to occur frequently.

In contrast, HGT by bacteriophages is frequent. The lifecycles of phages and bacteria resemble replication of retroviruses in eukaryotic cells, except that the majority of phages have DNA rather than RNA genomes. The viral DNA is integrated into the bacterial genome in “lysogenic phages”, which is equivalent to integrated DNA proviruses. The DNA proviruses received their name from the DNA prophages, which were discovered much earlier. Phage DNA genomes can also remain as episomal DNA plasmids inside lysogenic bacteria and be transferred to other bacteria by HGT or taken up as naked DNA. The phage genes can code for antibiotic resistance or toxins, or other new properties, such as increased virulence. We experienced this recently, when EHEC (entero-haemorrhagic *E. coli*) bacteria suddenly exhibited pathogenic traits and killed humans. Phages from animal feces supplied the toxin genes to the bacteria. Bacteria can pick up dozens of toxin or antibiotic resistance genes, which lead to multiresistant phenotypes against antibiotics. Again, many transferred genes may remain unnoticed. They are noticed most easily when causing diseases. Millions of phage genes have been sequenced and found to be unique, not present in the database and likely not derived from hosts. They are thought to be a major source of genetic diversity. Yet, many of the conserved phage genes seem to be unrelated to host genes [28]. Do they change so fast? One may have to examine the role of phages in HGT for the host-cell genomes in more detail.

Plants may also acquire novel genes by gene transfer, often not directly from viruses but indirectly via bacteria, which deliver tumor genes, such as the tumor-inducing Ti plasmids [82]. They are brought into the plant by wounds, where bacteria, such as *Agrobacterium tumefaciens*, can enter and supply the plasmid DNA, which we notice if cancer is caused. Insects or beetles can also transfer novel genes into plants. This gene transfer can be rather complicated and involve more than one step. In one well-studied case, it was shown that viruses can use fungi as intermediates to deliver genes to a plant. The virus-infected fungi grow in the roots of the plants and transfer the viruses. Their genes help the plant to survive extreme conditions such as dryness and heat [88]. Thus, the mechanism of HGT mediated by viruses could have been a major driver of growth and evolution of our and other genomes.

## Endogenization of retroviruses

The life cycle of retroviruses, as manifested today with available DNA-containing host cells, is unique in that the viral RNA genome can become part of the cellular DNA genome once the viral RT has made a DNA copy. Integration is an efficient survival strategy for retroviruses because the proviral DNA looks like cellular genes – it looks like “self.” It is inherited to each progeny cell as long as the cell lives and divides. An integrated DNA provirus is not easily recognized by cellular antiviral defense mechanisms.

**Fig. 5:**
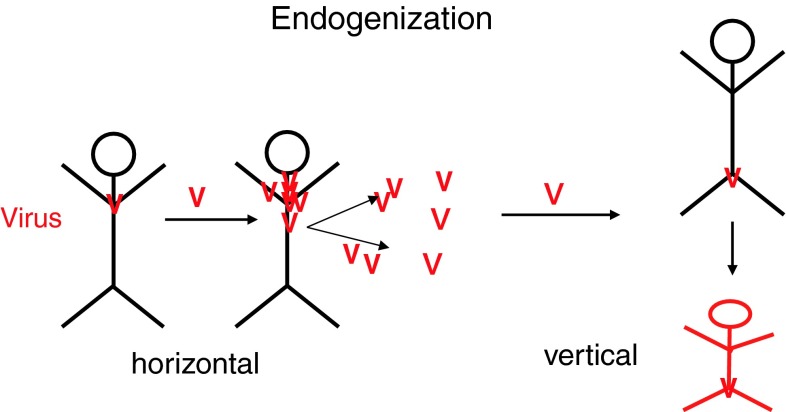
**Endogenization.** The endogenous retroviral elements in the human genome are attributed to previous horizontal retroviral infections. Retroviruses can in rare cases infect germline cells and be passed vertically to future generations. The host cell suppresses these genes during embryogenesis, but outside events can activate the viral elements to influence other genes. This happened to koalas in less than 100 years and made them resistant to the exogenous retrovirus. Integrated retroviruses, foamy viruses, can be dated back about 50 Mio years [53]. They do not cause diseases

Retroviruses have the unique ability to invade not only somatic cells but also the germline cells of their host, by endogenization. When the virus infects germ cells, it is transmitted vertically, from a mother to the progeny – in contrast to horizontal transmission by infection of somatic cells and other individuals [51] (Fig. 5). Germline cell infection is a danger that has to be avoided, also in gene therapy using replication-deficient retroviruses.

The human genome is full of transposable elements (TEs), endogenous viruses and various retroelements, which correspond to almost 50 % of the human genome [19, 59].

**Fig. 6:**
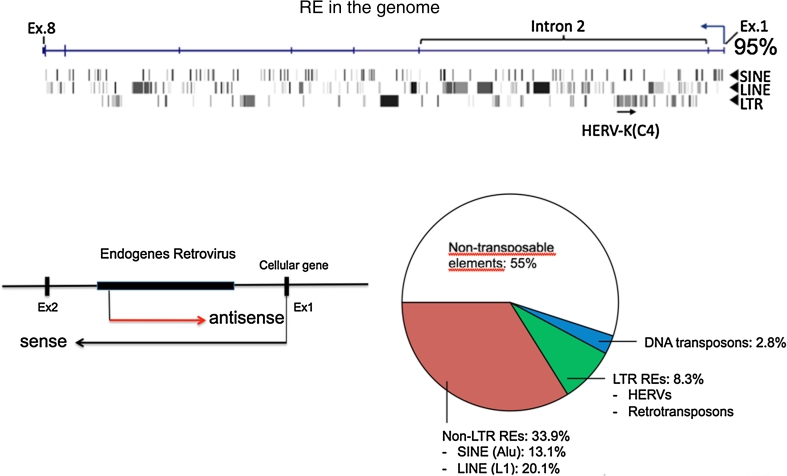
**Endogenous retroelements (RE). (Top)** A virus in a virus in a virus can be detected in cellular genes. One example is shown here with a protein kinase B inhibitor gene, which consists of up to 85 % REs [13, 59]. An integrated HERV-K(C4) is indicated. The inserts accumulate within introns, where integrations are less harmful than they would be in exons (Ex). REs comprise retroviruses and shorter versions, LINE, SINE or only LTRs [19]. **(Left)** The human endogenous retroviruses (HERV-K (C4)) can influence regulation of other genes, as shown for DAP3, a proapoptotic gene [13]. Antisense transcripts can shut off sense transcripts of a tumor suppressor gene and can thereby cause cancer. (**Right**) The number of human REs is shown as segments [modified from ref. 9]. The white area is investigated by ENCODE project

This was unexpected when it was discovered while sequencing the human genome [59]. On average, flies, worms and plants only contain 3-10 % transposable elements in their genomes [59]. Among the human TEs, there are about 450,000 retrovirus-like elements [59]. Infection of germline cells led to the accumulation of viral genes during evolution and made the human genome a “graveyard” of retroviral fossils. The host developed mechanisms to suppress such genes in the germline by silencing viral promoters through epigenetic modifications, e.g., DNA methylation. Mutations can likewise prevent protein expression and particle formation, yet these elements may affect host cell functions [51]. The significance of these TEs has been a fertile topic for speculation among biologists. Is it “junk DNA” [6]? Since this DNA can be transcribed into non-coding RNA (ncRNA) with gene regulatory function, it cannot simply be “junk.” The recent ENCODE (encyclopedia of DNA elements) project designated these regions as “deserts” – because of their lack of information, but important functions have now been suggested for these regions as well, especially for human diseases and gene regulation [7]. We also observed this when we studied a full-length human endogenous retrovirus HERV, which entered the human genome 35 Mio years ago, as calculated from the divergence between the two LTRs at the ends of the DNA provirus [13]. The viral promoter within the LTR is normally silenced by the antiviral response of the host cell. However, it can be activated by metabolic stress. If the activated LTR allows a transcript in the opposite direction to the transcript of a neighboring cellular gene, it is downregulated, and when it is expressed in parallel, it is upregulated. One such downregulated gene that we identified recently as a tumor suppressor gene is involved in apoptosis. LTR-driven antisense transcription resulted in prevention of apoptosis and cancer formation [13] (Fig. 6). Thus, 35-Mio-year-old endogenous HERVs can play a role in gene regulation and cancer formation to this very day. Only about 2 % of the human DNA codes for protein products [59]. This is only twice as much as in flies, worms and weeds. However, the genes are about 100-fold larger in humans, allowing complex regulation of gene expression, and with splicing, the number of coding genes is even higher [59]. The human genome contains about 40,000 HERVs today [19, 59].

The process of retroviral endogenization can be witnessed today. It was unexpected and observed by chance in Australian koalas. They were an endangered species in Australia and evacuated to offshore islands in the early 1900 s, where they contracted a monkey retrovirus, gibbon ape leukemia virus. Many of them died, but some that survived became resistant and showed endogenization of the virus into their germline [98]. It came as a surprise that this endogenization took only about 100 years, corresponding to 5 to 10 generations. For humans, with a 5-times longer doubling time, this would roughly amount to between 250 and 500 years. Is this a future scenario for HIV? Endogenization may have also occurred in SIV-resistant monkeys. How and when the monkeys developed host resistance genes to survive SIV is not known. Foamy retroviruses are non-pathogenic for both monkeys and humans. They co-evolved for about 100Mio years [53]. It has been suggested that some humans may have inherited antiviral resistance genes from survivors of smallpox from the Middle Ages. Progeny of the survivors of the Black Death may now be resistant to HIV, based on numbers and geographical distribution in Europe [38]. The 15 % of Caucasians who are genetically resistant to HIV infection today bear a mutation in a cellular receptor gene designated as Delta32 (31). Whether HIV can endogenize into human germ cells is controversial, because the germ cells may not have the receptors that would make them susceptible to infection by HIV.

## Viruses as builders of genomes

The DNA copies of retrovirus genomes accumulated in the host genome, where they are remnants of previous viral infections. They undergo mutations with time and acquire stop codons, deletions and insertions. Complex retroviruses with genes related to accessory genes are about 50 Mio years old, as shown for the foamy viruses, which are endogenous retroviruses [53]. However there is no reason to believe that no retroviruses existed or integrated before that. Some genes have more insertions than others, e.g., the human protein kinase inhibitor beta gene consists of about 85 % retroelements [59]. Many of them are nested integrations, one inside the other one, like a Russian doll. Removal of an integrated retrovirus by the host is difficult; suppression and mutations are alternatives. By homologous recombination between the retroviral promoters, the long terminal repeats or LTRs, the region between the two LTRs can be deleted. Then only one LTR is left from a full-length HERV. There are about 450,000 retrovirus-like elements, corresponding to 8.3 % of the total DNA, in the human genome (Fig. 6) [19, 59]. Completely different viruses have also become endogenous, which was unexpected. Ebola virus, bornaviruses and circoviruses have been found integrated in the human genome, even though they are RNA viruses, but they were integrated as DNA copies 50 Mio years ago [2, 52, 53]. Some illegitimate recombination events or reverse transcription may have allowed the integration of RNA viruses as DNA copies. Some of them still express proteins that may protect the host against infections. This seems to be the case for bornaviruses in humans, but not in horses, in which mental illnesses have been attributed to bornaviruses [2, 52, 53].

**Fig. 7:**
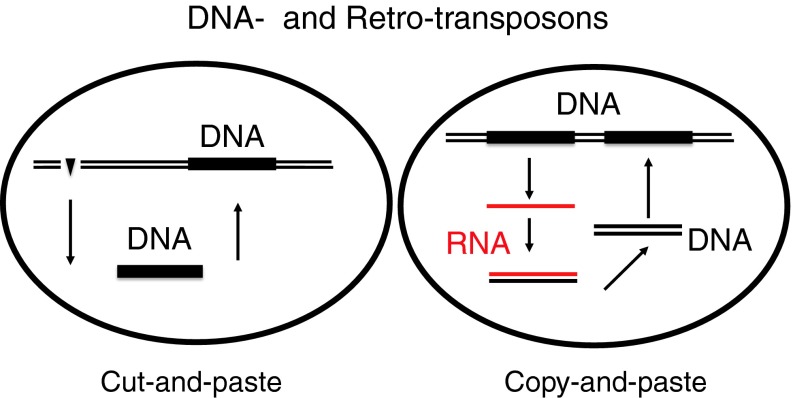
**Increase in the number of genes.** Genes can be cut out and pasted at another site in the genome, as described for transposons. This cut-and-paste happens actively in plants but ended in human genomes 35 Mio years ago [46, 81]. This leads to new phenotypes, depending on the site of integration, e.g., different colors in maize. A copy-and-paste mechanism requires an RT and increases the gene content of a genome, in 100 Mio years accounting for 20 % of the human genome [59]

Other retroelements are the LINEs, long interspersed nuclear elements. One subgroup is also named L1, and its members code for an RT, an integrase, an RNase H, and an RNA-binding protein, but they do not contain env or LTR promoter regions [19, 59]. Thus, they are incomplete retroviruses. There are about 850,000 copies of L1/LINEs, each about 6 kb in size. They cannot form particles and cannot leave a cell to infect a new one. Even though they are locked into a cell, they can be reverse transcribed, and the DNA can then be reinserted into the genome, thereby increasing its size, resulting at least in gene duplication. The mechanism is described as a “copy-paste” mechanism (Fig. 7) [59, 81, 89].

L1/LINEs can influence neighboring genes when mobilized and reinserted into genomes. They are found preferentially in introns, which is less damaging and allows the cells to survive better than if they were integrated into exons, i.e., the coding regions, which might result in a loss of those cells. L1/LINE1 have proliferated for 80 Mio years, affecting the human genome by generating mutations, genomic instabilities, alterations in gene expression, and genetic innovation [19, 59]. Integration of L1 elements took place with high frequency until 37 Mio years ago, for reasons that are not well understood [78]. Dramatic changes in the climate from meteorites or supernovae are being discussed as possible explanations. The L1 elements are twice as frequent in human genomes as in monkey genomes, and this might explain some of the differences between monkeys and humans. The L1 elements might lead to more complex gene regulation [59, 78].

Their contribution to mammalian genomes is probably underestimated because TEs have diverged beyond recognition. Most of them are inactivated by mutations so that they cannot replicate. They make up one third of our genome. About one hundred L1 elements are active today. The L1 elements can move around, as has been observed in the developing brain [78, 82, 91, 108] . There, they can deregulate gene expression and induce significant changes, causing diseases or special human traits. Again, diseases are very informative. In Rett syndrome, the L1 retrotransposons were found to have jumped much more frequently than in healthy brains [46, 62]. About 60 cases have been described with *de novo* insertions responsible for disorders such as haemophilia or cancer [46]. If this is not the tip of an iceberg, it is a rather small number in comparison to a whole genome. In other species, TEs are believed to have diversified the species, altered plants or insect-plant interactions, and helped in the development of flowers or odor in orchards to attract insects for gene exchange [105].

L1/ LINEs may be the most effective innovators in the genomes of many species.

Another group of non-LTR retrotransposons are the SINEs, small interspersed nuclear elements [19, 59]. They are short – a few hundred base pairs without protein-coding capacity – and use the help of LINEs for transposition. There are 1.5 Mio of them in the human genome. Alu elements, which are a few hundred nucleotides in length, are the most frequent SINEs in the human genome. They contain a recognition site for the restriction enzyme Alu, which gave them their name. They are the smallest and most successful TEs in the human genome, mobilized throughout the last 65 Mio years [19, 59]. They can influence transcription of flanking genes [19, 59].

Another very different class of elements are the DNA transposons [81]. They are not derived from retroviruses and do not code for an RT. They constitute about 2 to 3 % of the human genome [59]. DNA transposons can get in and out of the genome via a cut-and-paste mechanism and are designated as “jumping” genes [108]. DNA is cut out and subsequently integrated (pasted) at a different locus. The integration sites are random. Thus, the jumping genes or mobile elements can cause changes but do not result in an increase in the size of the genome as in the copy-paste mechanism (Fig. 7). Transposition is mediated by a transposase, coded for by the jumping gene itself, the DNA transposon. DNA transposons have been silent in the human genome for about 35 Mio years; however, they are active in plants [81]. Are they ancient and simple precursors of retroelements? DNA transposons were first recognized by B. McClintock [65] as non-Mendelian traits. She received the Nobel Prize in 1983, fifty years after her discovery. She would not have discovered this phenomenon in the human genome had she not analyzed colored maize, which has one of nature’s most dynamic genomes. It can lose and gain enormous amounts of genetic information – up to one third of its genome at a time. Plant genomes such as that of rice can be as large as the human genome, and 85 % of it can consist of DNA transposons [89]. DNA transposons are locked inside cells. Do DNA transposons have evolutionary relationships to DNA phages, which acquired a coat, making them able to leave and enter cells?

The human genome is unique in the sense that the size of one single gene coding for a protein can be as large as 100,000 base pairs [59]. We have about 20,000 genes, with protein-coding information accounting for about 2 %. Mice and rice have similar numbers of genes as humans, yet their genes are much smaller. In rice, one gene corresponds on average to 10,000 base pairs; in bacteria, to 1400; and in viruses, to about 1000. The genetic burden of the human genome is enormous. A large portion of it is due to TEs, of which we have about ten to twenty times more than other species [59]. This may lead to regulatory ncRNA, which may be left over from our RNA-dominated past [64, 83]. Thus, the human genome and regulation of gene expression involves sequences from our viral ancestors – as drivers of evolution.

## About the prevalence of DNA phages in bacteria and RNA viruses in plants

An important insight into the role of viruses in evolution may be gained from the phages. They are the most abundant and diverse entities on our planet and most successful in replication. We do not notice them because they do not normally cause diseases. They infect bacteria and are predominantly found in oceans (10^12^ /ml) and in the soil [97]. They are also present in our guts, on the skin, and in plants. Bacteria are ubiquitous, and so are their viruses [80, 85, 97, 101]. A striking property of DNA phages is their turnover. Eighty percent of bacteria are infected with phages, and every day about 20 % of all bacteria in the oceans are lysed through the activity of the phages, producing 50 % of the oxygen we need for breathing. Giant viruses can influence carbon metabolism, producing tons of carbon dioxide, which can influence our climate or contribute to rock formation, e.g., the White Cliffs of Dover [102].

Ninety-five percent of phages harbor double-stranded DNA genomes, linear or circular, of about 500 kb. The best-studied examples are the T4, T7 and lambda phages. Rare exceptions are single-stranded RNA phages, such as MS2 and Q beta, or circular single-stranded DNA phages, such as phi X174.

The dominance of DNA phages in the world indicates that they are very successful survivors. Phages extensively use the mechanism of HGT, e.g., of toxin or antibiotic resistance genes. They can thereby also increase bacterial host virulence [61]. DNA phages can either stay episomal or integrate into their bacterial host genomes while DNA viruses from mammals do not normally integrate in a mammalian host, perhaps because in contrast to bacteria, there is a nucleus as a barrier, blocking access to the host genome.

One may wonder why there are so few RNA phages and no known retrophages in the bacterial world. There are significant numbers of RTs in bacteria with some function [90, 109, 110], which may be indicators of a former retroworld. Also, certain DNA in bacteria is reminiscent of telomerases [90]. Thus, there are a few footprints of an earlier RNA or RNA-DNA world left in the DNA-dominated world of phages and bacteria today. Since the RNA and retroworld preceded the DNA world, the phages may have had RNA precursors, which they passed a long time ago and evolved further to a DNA world, replicating quickly, and that could be why DNA phages are so prevalent today.

In contrast to DNA phages, the plant viruses are almost always RNA viruses – and rarely, single-stranded DNA viruses – but almost never double-stranded DNA viruses. Thus, the question arises why one species is characterized by DNA virus genomes while others “maintained” RNA virus genomes. Since RNA preceded DNA, my speculative answer is that DNA phages have passed the RNA world because of their rapid replication rates, and RNA plant viruses appear to be closer to the RNA world, replicating much more slowly. Are they lagging behind? Even today viroids are “analphabets”, unable to code for proteins, and they are naked, not using coats. Thus, they appear to be close to the RNA and pre-protein world. Plant viroids may be precursors of plant viruses. Can’t we classify them also as viruses? Plant viruses are small, have no catalytic activity, and code for proteins – often only a few, in an economical way by exploiting two reading frames – which are used for simple structures. Thus, they appear ancient.

There is also a plant pararetrovirus, cauliflower mosaic virus, that harbors an RT – which is close to the retrovirus world. This example may suggest some evolutionary progress from RNA towards DNA, also in plants. Yet RTs may not have played such an important role in plants, which is suggested by indirect evidence, because plants do not activate RT-dependent retrotransposons as other eukaryotes do. Instead they use DNA transposons, which is a simpler mechanism. Today, DNA transposons actively jump only in plants, and active DNA transposons died out in other eukaryotes 37 Mio years ago [81]. Plants, especially maize and rice, have up to 85 % or even 90 % DNA active transposons, in contrast to humans, which have 3 % DNA inactive transposons, which are inactive [89]. Did DNA transposons evolve to retrotransposons? This is speculation, based on the thinking that simple mechanisms evolved to higher complexity.

Plants cannot move; they can only locally regulate their lifestyle. Active DNA transposons may help to deliver innovation. Insects, birds or the wind can spread plant viruses – possibly also as source of genetic innovation. Most surprising are plants such as rice, which has one of the largest genomes and can lose one third of it at a time [89].

The dichotomy between the DNA-dominated phages and the RNA-dominated plant viruses can perhaps be explained by considering the reproduction rates of their hosts, which influences the frequency of replication of their viruses. Bacteria replicate in about 10 minutes, depending on the nutrients available, while some plants can grow extremely slowly and are among the oldest species on earth, such as 3,500-year-old Sequoia trees. The reproduction time for humans of about 30 years ranges somewhere in between. Thus, bacteria may have replicated millions of generation times more than higher organisms. Evolution of bacteria and their phages may have progressed or evolved faster to a more DNA-dominated world. The RNA viruses in plants may have passed through fewer generations, and they are often inherited vertically with little genetic variation [88]. They seem to be more ancient.

Humans range in between these extremes. They are infected by a wide range of different RNA and DNA viruses, possibly reflecting ongoing viral evolution from RNA to DNA genomes. The regulatory ncRNA in our cells may remind us of our RNA past. DNA replication, telomeres and protein synthesis also strongly depend on RNA. Will the dominance of RNA in plant viruses be overcome by a more DNA-protein world sometime in the future after many more generations? Alternatively, plant viruses may be successful in plants as hosts and may be a dead-end branch in the tree of life.

## Strange viruses

Viruses are not simply pathogens, because most viruses never cause a disease. Viruses can have exotic properties: they can replicate in dead cells, repair radiation-damaged hosts, or recombine with other dead viruses and generate an intact cell [105–107]. In some cases, hosts can benefit from viruses [88]. An ancient retrovirus supported the development of the placenta in mammals. The virus HERV-W, an endogenous retrovirus, codes for an envelope protein, syncytin, related to the endogenous Jaagsiekte sheep retrovirus (enJSRV). It allowed the development of the human placenta by causing immunosuppression of the mother so that the embryo is not attacked by her immune system. Thus, a retrovirus-induced immunodeficiency was once of benefit for mankind [10, 68]. A later modification of this property may have led to the immunodeficiency we observe today with HIV. Viruses can transfer genes to plants to render them resistant to high temperatures [88].

An extreme example of mutualism between virus and host are the polydnaviruses (PDVs) [3,30,32,92]. In this case, the virus carries only host genes, and the host, a wasp, carries all of the viral genes – not vice versa, as one would expect. This exchange must be advantageous, since it is not even rare. PDVs are loaded with about 30 DNA host plasmids to protect and help feed the progeny in another environment. The virus survives as a DNA provirus in the genome of the wesp host, an endogenous virus that integrated 75 Mio years ago [32]. One may ask whether this is still a virus if it contains not a single viral gene inside its viral particle but only genes from the host. One would not have expected such a virus to exist – even though virologists have designed such viruses for gene therapy, probably without knowing of this invention of nature. The artificial viruses for gene therapy are degutted and filled with therapeutic genes against diseases and cannot replicate – just like PDVs [94]. There are many PDVs [3, 32, 34]. Thus, a “virus” lost all of its genes and became a mobile carrier of host genes. This phenomenon is not only observed for viruses: an extreme example among eukaryotes is the Elba worm. It has no mouth or gut and relies on outside bacteria for its food supply, digestion and recycling – thereby outsourcing all vital functions [27]. There is another surprise: a virus in a virus. The virophage Ma infects the giant virus CroV and integrates its genes into the viral host genome [33]. Also, bacteria became obligatory intracellular symbionts as mitochondria or chloroplasts of eukaryotic cells and delegated most of their genes to the nucleus, while the residual 300 genes, about 10 % of the total, were maintained and used for special functions. Is specialization traded off for loss of mobility [39, 66, 67]? Gene reduction or loss of genes may correlate with acquisition of special functions.

Do we have to redefine what a virus is? Several definitions of viruses have been summarized recently [76]. Many properties have been used as organizing principles of the virus world, such as symmetry and protein structure, the presence or absence of an envelope, the size of particles or genomes, RNA or DNA as genetic material, and replication rates or modes [1, 31, 48]. Is a virus a mobile entity of biomolecules that can replicate and evolve, that depends on some external energy source? Energy may not necessarily have to come from a host organism. Chemical energy could be taken from the environment. This is my definition, which resembles a recent proposal by NASA [76]. What about prions, which can replicate and mutate? They consist of proteins only. Prions can be found in most virology textbooks.

Are they a “self-sustained chemical system”? Mutation rates are almost a unifying parameter of all genomes – not only of viral but also cellular genes! (The mutation rate multiplied by the length of the genome equal one.) Too-high error frequencies caused by the application of mutagens can be detrimental and lead to “error catastrophe” [30]. A speculative approach would be to organize viruses according to their “age”, i.e., their suspected time of appearance during evolution, based on their replication rates, in the following putative timeline: viroids, RNA viruses, retro- and para-retroviruses, giant viruses, DNA viruses. The fast-replicating small DNA bacteriophages passed this developmental line more rapidly than the eukaryotic DNA viruses. DNA viruses from higher organisms are few in number compared to the DNA phages, and some RNA viruses such as the plant viruses may be closer to the beginning. There appear to be two threads of life: the bacterial thread, which I like to refer to as “fast, small, many”, and the eukaryotic thread, “slow, big, few”. Is there a continuum between these two extremes? It is worth noting but not surprising, that the bacterial-virus world is self-sufficient, because it existed long before us and other higher organisms. The microbes do not need the eukaryotes, but the eukaryotes need the microbes.

What came first, the virus or the cell? This question has stimulated an intense recent debate [54, 56, 76]. My speculative answer is: RNA viruses came first, then DNA viruses came, both ahead of cells. Cells are too complicated to be first (Fig. 4)! Retroviruses helped to build and shape cellular genomes. Did some viruses evolve to become giant viruses? They can even be infected by a virophage with phage genes integrated into the giant viral genome [33]. Giant viruses may indicate that the borderline between viruses and cells is transient, but this is controversial [76]. Then viruses would be our oldest ancestors!

## Co-evolution of viruses and antiviral defense

**Fig. 8:**
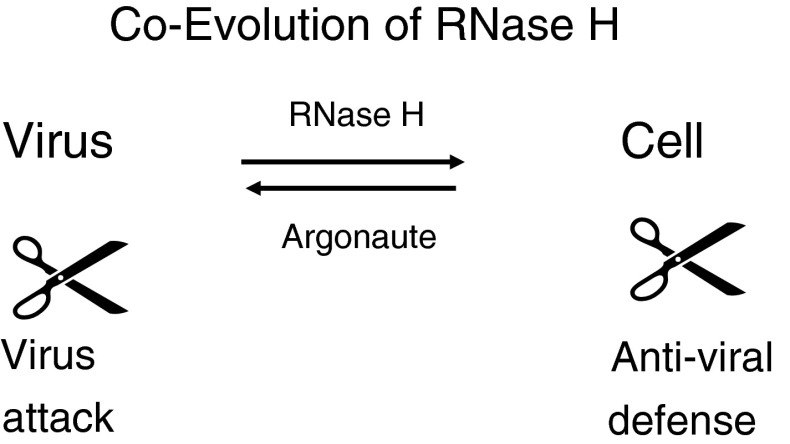
**Coevolution of RNase H**. Viruses and hosts harbor similar elements. Viruses can provoke antiviral responses. The viral and host elements coevolve. A striking example is the composition of a retrovirus, which is analogous to that of the antiviral defense machinery [70, 72]. The RNase H is structurally related to the enzyme component of Argonaute of the small interfering siRNA machinery. The components are similar, and the enzymes involved are structurally closely related, which cannot be detected at the primary-sequence level

Coevolution between retroviruses and cells resulted in a surprising phenomenon: similarities between viruses and host anti-viral defense machineries. Each component of the virus has an equivalent in the cellular antiviral defense system [72]. The retroviral RT with the RNase H is homologous to PAZ and PIWI, components of the defense enzyme Argonaute 2, which mediates RNA silencing [70]. This is not easily detected by comparing primary sequences but it is striking in crystal structures. RNase H and PIWI have crystal structures and enzymatic functions that are very similar to those of nucleases [70, 72] (Fig. 8, scissors). Thus, structures are more highly conserved than sequences. Other components of the virus and the siRNA silencing defense system are similar as well, indicating significant co-evolution of most or even all components of retroviruses and cells [70, 72]. Did viroids and antiviral microRNAs, miRNAs, coevolve? This is suggested by similarities of the terminal parts of the viroids resembling the defense miRNAs [100]. Such similarities appear as logical consequence of coevolution. The antiviral cellular defense systems also reflect viral properties in other cases. The siRNA-based immune system is an antiviral defense against RNA viruses, which was discovered in plants. The bacterial defense or “CRISPR” system, directed against DNA phages, is DNA-based [92]. DNA of the invading phage is stored and remembered in case of a novel infection. Bacteria then produce RNA against the invaders as a defense. It requires an RNase H – as described above for retroviruses and antiviral defense. Thus, there are RNA-, DNA- and protein-mediated antiviral defense mechanisms – apparently reflecting stages of evolution from RNA to DNA to proteins [63, 72]. With the help of retroviruses, CRISPR may have even evolved further, into the immunoglobulin rearrangements in mammals [49]. Recent studies suggest that, instead of constantly fighting, phages and bacteria in our guts are normally in a well-balanced state of equilibrium [40, 85].

Only when things go wrong, such as when natural hosts are lost due to changes in environments, will viruses leave well-balanced equilibrated situations and infect naive new or weak hosts and cause diseases. Most of them are man-made accidents exploited by opportunistic viruses.

## An outlook - phages as promise?

Phage research is undergoing a renaissance, not with respect to basic research on gene regulation but with respect to health and biotechnology. Phages killing bacteria had been used for almost 100 years as antibacterial therapy before the discovery of antibiotics. Phage cocktails were utilized in the Soviet Union in the Russian-Finnish war to avoid amputations on injured soldiers. Phage therapy is currently not fashionable in Western medicine, although it may gain attention again if resistance of bacteria to antibiotics continues to grow and increase the danger for human health [95]. A stool transfer, replenishing the whole microbiome with a single treatment, can be a life-saving procedure for critically ill patients. It is cheap, efficient and risk-free. We analyzed the microbiome of such a patient after transfer [28], who recovered within a few weeks. It was composed of microbiomes of donor and recipient, and it changed over time (Moelling et al., unpublished results). Perhaps one can fight obesity by a microbiome replacement or phage cocktail or in the future by a new kind of phage pill – this is speculation. One can already order phages as a personalized therapy from Tbilisi, Georgia. A large Swiss pharmaceutical company is using T4 phages therapeutically. Historically, rivers like the Ganges were thought to cure infectious diseases, e.g., cholera or leprosy. That capacity was later attributed to phages present in the river. Thus, there is a scientific basis for this religious ritual. Phages are being sprayed on potatoes to prevent their decomposition or as a preservative of other nutrients, including milk. Phages are being tested to kill the blight fungus, which threatens many chestnut trees. Phage-treated food may be more acceptable to the public than genetically modified food because it is a transient treatment, not a gene-modifying procedure, and can be selected to be harmless.

Can we learn from phages or viruses about our future? Phages influence population dynamics. The interaction between phages and bacteria is stress-dependent, caused by a lack of food or space. If nutrients become scarce, the phages lyse the bacteria, reduce bacterial growth and recycle nutrients, and giant viruses lyse algae, which prevents overgrowth [102, 103]. After lysis nutrients will be recycled and life will recover. So, the viruses give us some hope. Viruses may regulate population dynamics for humans as well. HIV and influenza pandemics may already be warning us what might happen to mankind in a future with megacities and high population densities.

Will the viruses and microbes survive humans when mankind runs out of nutrients? Bacteria, archaea and viruses can adjust rapidly to environmental changes, while humans cannot. Who will be the fittest survivors – humans, insects, cockroaches, plants, bacteria, viruses, or archaea? Archaea have adjusted to extremes in the past. Will they find a niche in the future? We cannot survive without viruses and bacteria, which is a surprise. This is new and in contrast to our historical view of viruses and bacteria as pathogens. Viruses and epidemics can even affect social behavior as an evolutionary force. This may have been true for mankind ever since its beginning, when coping with infectious diseases just like enemies [15, 69].

Can there be exoplanetary life? The mission of the spaceship Curiosity is to find life on Mars. Is it being hampered by non-sterile equipment on board the spaceship? It was very surprising when the Apollo 12 spaceship came back from the moon containing spores from the earth that had been in orbit for two years, but regrew despite exposure to radiation, vacuum and extremely low temperatures. Life can even regrow from spores from bacteria that were inside insects locked in amber for 25-40 Mio years [16]. A 35 Mio-year-old HERV was reconstructed from several defective HERV sequences by deduction of a consensus sequence as infectious virus, designated Phoenix [24].
